# Obesity Prevents S-Adenosylmethionine-Mediated Improvements in Age-Related Peripheral and Hippocampal Outcomes

**DOI:** 10.3390/nu13041201

**Published:** 2021-04-06

**Authors:** Jacob W. Vander Velden, Danielle M. Osborne

**Affiliations:** R.S. Dow Neurobiology Department, Legacy Research Institute, Portland, OR 97232, USA; jvandervelden1@student.gsu.edu

**Keywords:** aging, anxiety, s-adenosylmethionine, transforming growth factor b-1, TGFβ1, obesity, microglia, glutathione

## Abstract

Background: Age predisposes individuals to a myriad of disorders involving inflammation; this includes stress-related neuropsychiatric disorders such as depression and anxiety, and neurodegenerative diseases. Obesity can further exacerbate these effects in the brain. We investigated whether an inexpensive dietary supplement, s-adenosylmethionine (SAMe), could improve age- and/or obesity-related inflammatory and affective measures in the hippocampus. Methods: Mice were placed on their diets at six weeks of age and then aged to 14 months, receiving SAMe (0.1 g/kg of food) for the final six weeks of the experiment. Prior to tissue collection, mice were tested for anxiety-like behaviors in the open field test and for metabolic outcomes related to type 2 diabetes. Results: SAMe treatment significantly improved outcomes in aged control mice, where fasting glucose decreased, liver glutathione levels increased, and hippocampal microglia morphology improved. SAMe increased transforming growth factor β-1 mRNA in both control mice, potentially accounting for improved microglial outcomes. Obese mice demonstrated increased anxiety-like behavior, where SAMe improved some, but not all, open field measures. Conclusions: In summary, SAMe boosted antioxidant levels, improved diabetic measures, and hippocampal inflammatory and behavioral outcomes in aged mice. The effects of SAMe in obese mice were more subdued, but it could still provide some positive outcomes for obese individuals dealing with anxiety and having difficulty changing their behaviors to improve health outcomes.

## 1. Introduction

Obesity is a chronic inflammatory disease; alone it perturbs metabolic function, but also can exacerbate the negative effects of aging on the brain, and more specifically the hippocampus [1]. Age and obesity are both demarked by steady increases in several pro-inflammatory factors, effects largely induced by white adipose tissue driving activation of pro-inflammatory kinases (i.e., JNK, NFκB, and IκB kinase β reviewed in [2,3]) that target insulin-sensitive organs. The brain is an insulin responsive tissue [4] that is susceptible to inflammatory damage [5] by kinases known to be increased by aging [3], and made worse by obesity [6], and which activate the brain’s immune cells, microglia. Activated ameboid-shaped microglia are triggered by metabolic dysfunction and implicated in hippocampal pathological conditions [7] and impact surrounding neurons by releasing cytokines (reviewed in [8]). Key to controlling microglia is transforming growth factor beta-1 (TGFβ1). TGFβ1 is necessary for maturating microglia, while TGFβ1 deficient mice are completely void of brain microglia [9]. Neurodegenerative diseases suppress TGFβ1 leading to perturbed microglial homeostasis [10], supporting that decreased TGFβ1 can indicate declining brain health and activated microglial states. Thus, therapeutics that safely reduce activated microglia may help alleviate symptoms of neurological conditions.

S-adenosylmethionine (SAMe) is an over-the-counter (OTC) dietary supplement and is sought for its ability to increase levels of the antioxidant glutathione [11]. SAMe is decreased in the livers of non-obese type 2 diabetic Goto-Kakizaki rats [12], db/db mice [13], and mice fed a high-fat diet [14]. Inclusion of SAMe into a diet attenuated sucrose-facilitated meal-induced insulin sensitization, an indicator of diabetic development, possibly due to its ability to increase glutathione in the liver [15]. SAMe plasma levels are also decreased in Type 2 Diabetic patients, while those with the lowest SAMe also demonstrated mild cognitive impairment [16]. SAMe has other demonstrated abilities to affect the brain. Cultured microglia treated with SAMe showed attenuated levels of LPS-induced increases of IL-1β, TNF-1α, and IL-6 gene expression [17]. Several studies have demonstrated cognitive and pathological improvements in Alzheimer’s disease transgenic mouse models treated with SAMe [18,19,20,21]. Although SAMe has shown promise in clinical and pre-clinical trials for ameliorating cognitive decline and depressive symptoms [22], it has not been evaluated for anxiolytic/anxiogenic effects, another behavior with a strong link to hippocampal function and a common co-morbidity among obese [23], aging [24], and/or demented patients [25]. We hypothesized that feeding aged mice SAMe, with or without diet-induced obesity (DIO), would improve peripheral measures of insulin resistance, decrease inflammation in the hippocampus, and improve affective behaviors in both aged and obese aged mice. Results supported that SAMe has the potential to improve blood glucose regulation, increase hippocampal TGFβ1 levels, reduce reactive microglia, and improve affect; however, the effects in obese mice were more limited.

## 2. Materials and Methods

All methods and procedures performed on mice were approved by the Institutional Animal Use and Care Committee at Legacy Research Institute and conducted under protocol LRI-111-2017. Mice were maintained on a 12/12 h light/dark cycle, with lights on at 0730.

### 2.1. Mice and Diets

C57BL/6 male mice were obtained from Jackson laboratories at five weeks of age and housed 5/cage. Mice were randomly assigned to either control chow (Chow, Purina 5001) diet or diet-induced obesity (DIO) using an obesogenic diet, 60% high-fat food +10% sucrose in the water, beginning at 6 weeks of age. Mice had ad libitum access to food and water and were handled regularly for the duration of the experiment when they were weighed/health checked weekly. SAMe administration was based on previously published methods, which also found this dose increased brain levels of SAMe [19]. At 12 months of age, half the mice were given a modified version of their existing diet containing 0.1 g of SAMe/kg of food. Mice consumed approximately 400 μg of SAMe/day based on 4 g of food consumption/mouse/day. SAMe was added to a standard 60% high-fat diet (D12492, Research Diets) or a custom control diet formulated with the same macronutrient content as Purina 5001. SAMe (derived from Denosyl 425 mg) was obtained from a commercial source. Special diets were maintained until tissue was collection at 14 months of age. Mice were euthanized by rapid cervical dislocation followed by decapitation. Blood was collected and stored on ice until it was centrifuged and the supernatant removed and stored in a separate collection tube. Samples were stored at −80 °C. The dorsal hippocampus was bilaterally dissected out, the rest of the brain was kept, and a small piece of liver was removed. Samples were immediately frozen in liquid nitrogen and stored at −80 °C. 

### 2.2. Glucose Tolerance Testing (GTT)

Prior to SAMe treatment, as a means to determine the extent of metabolic dysfunction, mice were tested for insulin resistance at 9 months of age to confirm phenotype. Mice were fasted for six hours beginning at lights on at 0730, but maintained ad libitum access to water (sucrose water was replaced with regular tap water in DIO mice). Mice were then administered 1.5 g/kg i.p. of dextrose. Whole blood was tested using AgaMatrix Presto meter and testing strips at baseline and 15, 30, 60, 90, 120, and 180 min post-dextrose. At completion of the final time point, mice were returned to their homecage with reinstated ad libitum access to food and water. 

### 2.3. Open Field 

Mice were placed into the periphery of the open field box (40 cm × 40 cm × 40 cm) and allowed to freely explore for 30 min. Speed and distance were analyzed using Ethovision Software (Noldus, Wageningen, The Netherlands) in five minute bins, from a ceiling-mounted camera. Anxiety-like behavior was determined based only on the first 10 min in the box by measuring the latency to enter, and the duration of time, spent in the center of the open field box.

### 2.4. Immunohistochemistry

Mice (*n* = 5–8/group) were put under anesthesia with isofluorane. Ice cold PBS was perfused through each animal followed by ice cold 4% paraformaldehyde. Brains were removed and placed in 4% paraformaldehyde for 24 h, then were transferred to 30% sucrose until they sunk, and then stored at −80 °C. Brains were sectioned at 40 microns. Sections were permeabilized in 1% Triton and PBS for 1 h followed by 2N HCl for 30 min at 37 °C then neutralized with 0.1 M sodium tetraborate and incubated for a further 10 min at room temperature. Slices were blocked with appropriate goat donkey blocking buffer for 1 h at room temperature then primaries were added for overnight incubation at 4 °C on rotator: Iba1 (Abcam, 1:500). The next day slices were rinsed then incubated with appropriate secondary antibody (AlexaFluor, Life Technologies) for 1 h at room temperature. Dapi was applied in the coverslip mounting medium. Slides were imaged by confocal imaging Leica LasX software system (Leica, Buffalo Grove, IL, USA). 

Microglia size measurements were completed by Leica Application Suite analysis software (Leica, Buffalo Grove, IL, USA) and Image J. Images were taken at 40x with a resolution of 2048 × 2048 for maximum visibility centered on the CA1. All scoring was completed by experimenters blind to conditions and based on previous methods [26]. For soma measurements, Leica software was used to remove the background and a frame (1200 × 1000 px; 153,938 μm^2^, this same sized frame was used throughout for all morphological measurements) was placed within the CA1. Only microglia completely within the frame were included in analyses (31–90 microglia in each image). Although the software isolated microglia, every image was carefully scanned and compared with the original to ensure whole visible microglia were included. For branch counting, ImageJ software was used to create a skeletonized binary high-contrast image (Skeletonize3D plugin) to isolate microglia (20–24 cells/image) and analyzed using the AnalyzeSkeleton plugin. 

### 2.5. qPCR

All qPCR procedures were conducted by the Gene Profiling Shared Resource Center at OHSU (Portland, OR) using largely automated methods. RNA was isolated from hippocampus (*n* = 4–5/group) using Qiagen RNeasy mini kit with QIAcube automation. RNA quality was assessed by Agilent 2100 Bioanalyzer with a Eukaryote total RNA Nano chip. The core also performed reverse transcription using SuperScript VILO cDNA synthesis kit (Invitrogen, #11754050) with ~16 ng RNA per well. Following cDNA synthesis, 2 μL of cDNA was used in the PCR reaction using 10 μL of TaqMan Master Mix II (Invitrogen, #4440040) and 1 μL of 20× gene specific TaqMan assay was loaded into QuantStudio Real-time PCR System (Life Technologies) using a single mastermix per TaqMan probe for Transforming Growth Factor beta 1 (TGFB1; Mm01178820_m1, ThermoFisher). Data was collected using Applied Biosystems QuantStudio 12K Flex Software (v1.2.2). GAPDH (Mm99999915_g1, ThermoFisher) was used as the control gene in calculating ΔCT. Ct levels were all within the acceptable range, reference gene variance was stable across the samples, and replicates showed inter-assay validity. 

### 2.6. Glutathione Assay

Measurement of reduced glutathione in brain and liver utilized a kinetic assay kit (Sigma #CS0260) and followed manufacturer’s instructions (*n* = 7–8/group). In brief, several hundred milligrams of tissue was required for this ELISA: liver and whole brains (minus hippocampus used in qPCR) were dissected from mice following cervical dislocation. Samples were immediately placed in liquid nitrogen and then transferred to −80 °C for long-term storage. For preparation, samples were pulverized, weighed (10–300 mg required for assay) and then 3 volumes of 5-sulfosalicylic acid added and vortexed, and another seven volumes of 5% SSA solution added. Samples were then homogenized with a pestle in a glass tube until an even suspension was achieved. Samples were left on ice for 10 min prior to centrifuging at 10,000× *g* for 10 min. The volume of the supernatant was measured. Samples were further diluted 1:20 with 5% SSA to obtain the working solution. Blanks, standard curve and samples were plated (samples were counterbalanced across conditions and organ type). Following manufacturer instructions, the plate was read in a plate reader, measuring absorption at 412 nm at one-minute intervals for five minutes. All samples were run in triplicate. 

### 2.7. S-Adenosylmethionine ELISA

SAMe was measured in serum samples per manufacturer instructions (BioVision, E4541). In brief, serum samples were diluted 1:1 with provided sample buffer and run along with standards in duplicate. Plated samples were combined with biotin-detection antibody and allowed to incubate for 45 min at 37 °C. Solution was removed, and the plate washed three times with 1× wash buffer. HRP-streptavidin conjugate was added to the wells and incubated for a further 30 min at 37 °C. Plate was emptied, and then washed 5 times with 1× wash buffer. TMB substrate was added to the wells and incubated for 15 min at 37 °C, at which point STOP solution was added to terminate the reaction. Plate was immediately read at 450 nm.

### 2.8. S-Adenosylhomocysteine Hydrolase ELISA

S-adenosylhomocysteine hydrolase (SAHH) was measured in serum samples per manufacturer instructions (Aviva Systems Biology, Ahcy ELISA kit (Mouse), OKEH03618). In brief, serum samples were diluted 1:3 with provided sample buffer and run along with standards in duplicate. Samples were plated and incubated for 2 h at 37 °C. Samples were removed, and 1× biotinylated Ahcy detector antibody was added to the plate and incubated for 1 h at 37 °C. Fluid was removed and the plate was washed three times with 1× wash buffer. Avidin-HRP Conjugate was added to each well and the plate incubated for 1 h at 37 °C. Fluid was removed and the plate was washed five times with 1× wash buffer. TMB solution was added to the wells and incubated for 22 min at 37 °C, at which point STOP solution was added to terminate the reaction. The plate was immediately read at 450 nm. 

### 2.9. Experimental Design and Statistical Analysis

Two-way ANOVAs were conducted for all comparisons (Diet x SAMe Treatment), except for GTT open field distance which were mixed model repeated measures ANOVAs. Statistical analysis and graphing was completed using GraphPad Prism 9.0 Software.

## 3. Results

### 3.1. SAMe Improves Fasting Glucose in Chow, but Not DIO, Mice

**Figure 1:**
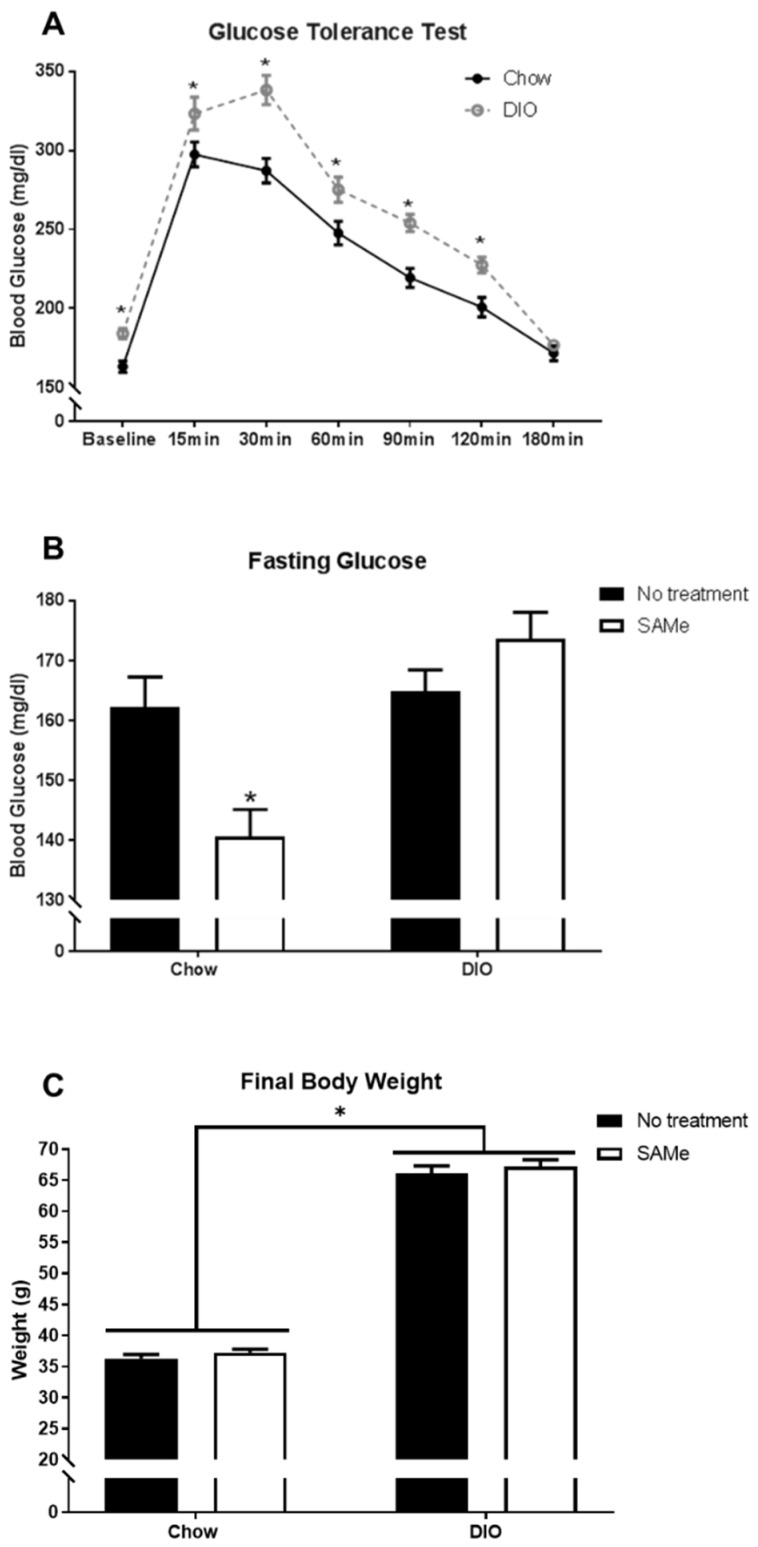
(**A**). Diet-induced obesity (DIO) mice had significantly impaired glucose regulation following glucose tolerance test (*n* = 43–53/group). (**B**). Following six hours of fasting, Chow mice fed SAMe demonstrated improved glucose homeostasis with reduced blood glucose levels relative to all other condition groups (*n* = 18–25/group). (**C**). DIO mice weighed significantly more than mice fed a Chow diet, regardless of SAMe treatment. *: Significant difference.

GTT was done prior to the administration of SAMe to confirm metabolic dysfunction in DIO mice. DIO mice developed insulin resistance as indicated by significantly increased and prolonged blood glucose levels following dextrose administration (significant interaction, F6,564 = 4.3, *p* < 0.001; Figure 1A). With SAMe supplementation, Chow mice showed a significant reduction in fasting glucose levels, an effect not observed in DIO mice (interaction, F1,83 = 11.7, *p* < 0.005; Figure 1B). There was a main effect of diet, where DIO mice weighed significantly more than Chow mice, regardless of SAMe treatment (F1,85 = 980.9, *p* < 0.0001; Figure 1C).

### 3.2. Chow, but Not DIO, Mice Show Improved SAMe Utilization with SAMe Treatment

**Figure 2:**
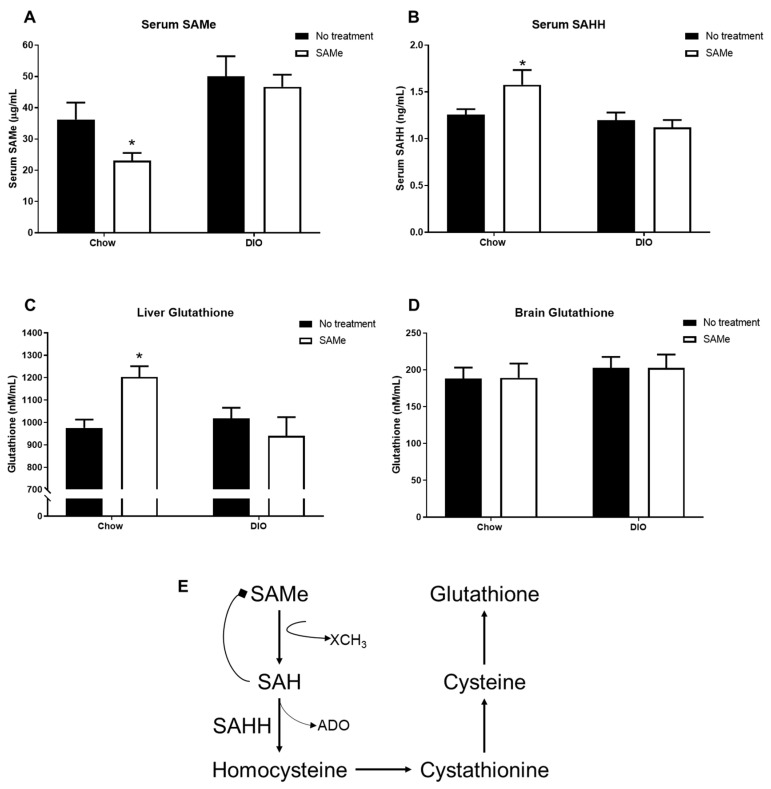
SAMe (**A**) and (SAHH) (**B**) were analyzed in serum samples (*n* = 10/group). Liver (**C**) and brain (**D**) were collected for analysis of total glutathione levels (*n* = 7–8/group). In Chow mice only, SAMe treatment lowered serum SAMe, but increased serum SAH and liver glutathione. (**E**) Summary of key aspects of the transmethylation and transsulferation pathways that pertain to SAMe, SAHH, and glutathione levels. *: Significant difference.

Chow + SAMe mice had significantly decreased serum levels of SAMe (F1,36 = 15.2, *p* < 0.001; Figure 2A), while SAH levels were significantly increased (F1,36 = 6.5, *p* < 0.05; Figure 2B). Chow mice treated with SAMe showed a significant increase in liver glutathione levels, relative to other groups, while no change was observed in DIO mice (interaction, F1,26 = 7.7, *p* < 0.05; Figure 2C). No changes in brain glutathione levels were detected in either Chow or DIO mice (Figure 2D).

### 3.3. DIO and SAMe Treatment Affected Measures of Microglial Activity in the CA1 Region of Dorsal Hippocampus

**Figure 3:**
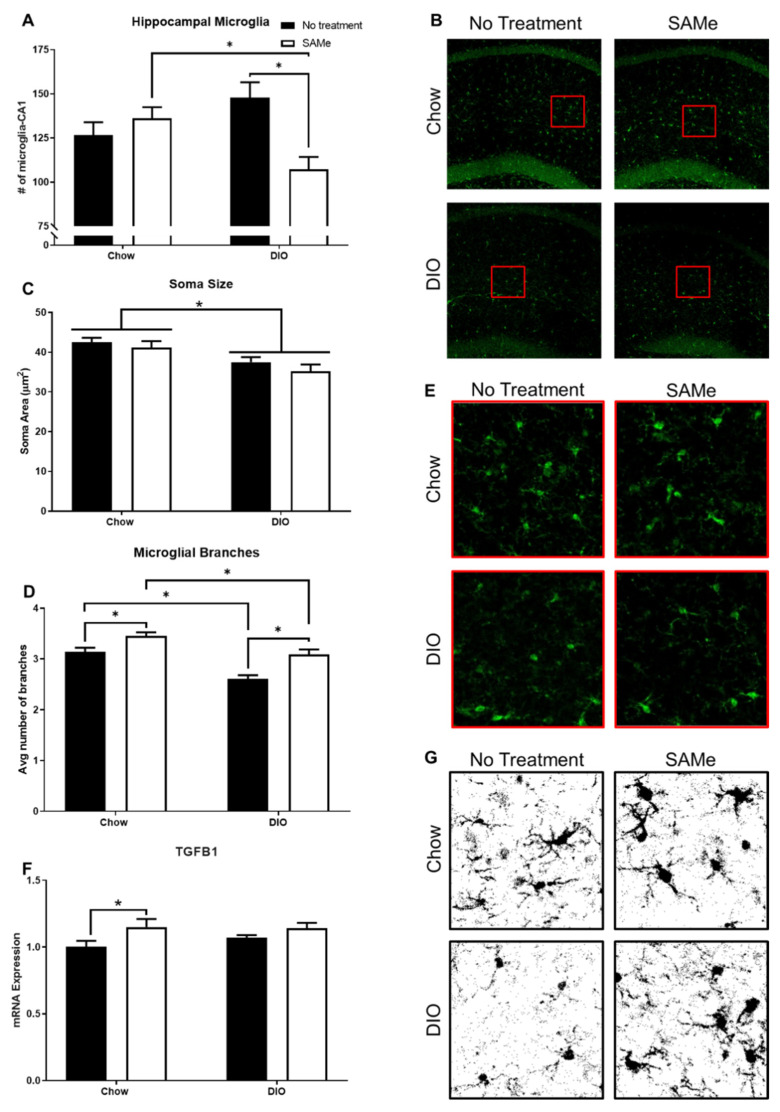
Hippocampal microglia were quantified within the sub-pyramidal area of CA1 for each condition (*n* = 5–7/group). (**A**) SAMe treatment in DIO mice significantly reduced microglia density within the CA1, compared to Chow + SAMe and untreated DIO counterparts. (**B**) Representative confocal images of hippocampal microglia. (**C**) Somal area of microglia were measured. DIO significantly reduced the cell body size of microglia. (**D**) Representative confocal images of microglial morphology. (**E**) Both DIO and SAMe affected the number of microglia branches. (**F**) Representative high contrast images. (**G**) Transforming Growth Factor beta 1 (TGFb1) was measured by qPCR (*n* = 4–5/group) using hippocampal samples. SAMe treatment significantly increased TGFb1 expression in Chow mice. *: Significant difference.

DIO negatively affected microglia soma area and branching, but not density or TGFB1 transcripts; SAMe improved branching outcomes of both Chow and DIO mice, while also affecting microglial density in DIO mice and TGFB1 transcripts in Chow mice only. SAMe treatment to DIO mice decreased the number of hippocampal microglia compared to Chow + SAMe and DIO + No Treatment mice. There was a significant interaction (F1,19 = 11.8, *p* < 0.01; Figure 3A,B) and main effect of treatment (F1,19 = 4.5, *p* < 0.05) in the number of microglia counted within the sub-neuronal area of CA1 region of the dorsal hippocampus. Morphologically, DIO mice had significantly smaller microglial bodies, commensurate with a heightened inflammatory state expected from obesity (significant main effect, F1,22 = 13.3, *p* < 0.01; Figure 3C,D). There were significant main effects for diet (F1,22 = 29.4, *p* < 0.0001) and treatment (F1,22 = 23.5, *p* < 0.0001); DIO mice had significantly fewer microglial branches, while SAMe treatment increased branching in both Chow and DIO mice (Figure 3E,F). Finally, there was a main effect of SAMe treatment (F1,15 = 6.1, *p* < 0.05; Figure 3G) on TGFB1 transcripts. Post hoc tests demonstrate that SAMe increased transcripts of TGFB1 in the hippocampus of Chow mice only, no differences relative to untreated DIO mice were observed.

### 3.4. SAMe Treatment Ameliorates Some of the Increases in Anxiety-Like Behavior Caused by DIO

**Figure 4:**
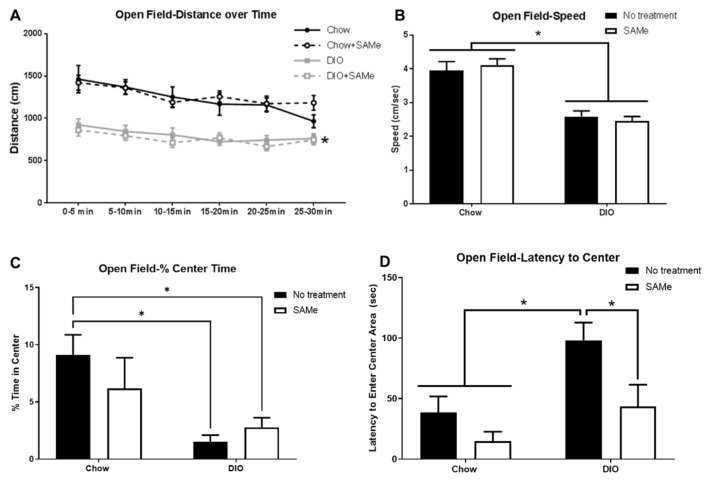
(**A**) DIO mice placed in the open field were less mobile than their Chow counterparts (*n* = 12–15/group), there was no effect of SAMe. (**B**) DIO mice did not move as quickly as Chow mice, SAMe did not affect speed. (**C**) DIO mice spent a lower percentage of their time in the center of the open field than untreated Chow mice. (**D**) SAMe treatment improved latency to enter the center area of the open field in DIO mice. *: Significant difference.

Not surprisingly, DIO mice covered less distance in the open field, importantly though, they moved consistently throughout the entire testing period (main effect of diet, F3,50 = 21, *p* < 0.0001; Figure 4A), albeit at a slower pace than Chow mice (main effect of diet, F1,50 = 62.5, *p* < 0.0001; Figure 4B). DIO mice also demonstrated increased anxiety-like behavior with lower percentage of time spent in the open field center (F1,24 = 6.8, *p* < 0.05; Figure 4C) and in greater latency to enter the center area (main effect of diet: F1,24 = 7.6, *p* < 0.05), with the latter effect showing amelioration with SAMe treatment (main effect of treatment: F1,24 = 6.0, *p* < 0.05, Figure 4D).

## 4. Discussion

The body operates poorly under obese conditions. In particular, when compounded by age, hippocampal functions can become impaired [27,28]. We looked at age-related markers of peripheral and hippocampal health, with or without the addition of long-term obesity, and determined that SAMe supplementation is beneficial to aged mice across all measurements, but the positive effects of SAMe were diminished with obesity. Non-obese mice showed greater benefits from SAMe, with a reduction in fasting glucose and increased glutathione levels in the liver, while in their brains, TGFβ1 levels were increased, and microglial morphology improved. Obese mice however, showed no improvement in peripheral markers, and only a slight improvement in some anxiety-like behaviors, a reduction in hippocampal microglia and improved branching with SAMe administration. Ultimately, SAMe demonstrated promising ability to attenuate age-related dysfunction in the periphery and hippocampus, but those effects are attenuated by obesity.

Contrary to the expectation that SAMe would have more ubiquitous effects, SAMe supplementation decreased fasting glucose levels in Chow mice only. With obesity, and even with advanced aging [29], glucose homeostasis becomes perturbed, as tissues become insulin insensitive leading to less glucose uptake and more endogenous glucose production [30]. The liver in particular mediates endogenous postprandial glucose creation from its glycogen stores [31]; in diabetics, the liver releases excessive glucose leading to persistent hyperglycemia [30]. The improved glucose measure in Chow + SAMe mice may reflect improved liver function, as is supported by the increase in SAHH and liver glutathione that was observed exclusively in Chow + SAMe mice. 

The elevated glutathione is likely a product of improved and increased SAMe metabolism through the transmethylation and transsulferation pathways (Figure 2E). SAMe is metabolized by NNMT into s-adenosylhomocysteine (SAH). As SAH is a potent inhibitor of SAMe-mediated methylation reactions [32], and its accumulation often coincides with hyperhomocysteinemia, SAHH is the only identified enzyme capable of SAH hydrolysis, making it vital to maintaining cellular functions [33]. The decrease in serum SAMe in Chow + SAMe was unexpected, however, with elevated SAHH, it supports that supplemental SAMe to Chow mice can enhance the efficient metabolism of SAMe, and ultimately increase glutathione. Fuso et al. [19] administered SAMe for three months and did observe a significant change in plasma SAMe levels. The dietary delivery methods for this study were based on their design; however, their study utilized three week old mice and a longer duration of SAMe delivery. The significant age difference (3 months vs. 14 months) and the shorter administration period (12 weeks vs. 6 weeks) may account for the differing plasma results in control mice. DIO mice demonstrated an inability to benefit from SAMe supplementation the same way as Chow mice. The insulin-sensitizing drug Metformin has been shown to interfere with H19 long noncoding RNA-mediated inhibition of SAHH [34], suggesting that DIO mice may be incapable of regulating SAHH activity via this AMPK-dependent mechanism [34]. DIO diets were not deficient in any dietary co-factors vital in SAMe metabolism, as such, the lack of changes to serum SAMe, SAHH, or liver glutathione levels with SAMe supplementation reflects a general inability to take up nutrients and perform basic metabolic functions within DIO mice. Obesity, and the non-alcoholic fatty liver disease (NAFLD) and more severe nonalcoholic steatohepatitis, that accompanies it, is associated with severe dysfunction of glutathione-related enzymes [35,36] and inhibition of SAHH activity [37]. Conversely, genetic deletion of glutathione s-transferase pi-isoform in mice results in significantly reduced glucose tolerance, increased JNK-mediated inflammation, and increased hepatic gluconeogenesis; additionally, the high-fat diet mice were even more susceptible to glutathione manipulations [36]. Although we do not know whether insulin resistance was improved due to the lack of post-SAMe GTT results, the increase in liver glutathione in Chow mice may have been sufficient to decrease age-related increases in gluconeogenesis, thus lowering fasting glucose levels, while DIO mice experience a much more severe level of liver dysfunction and probable NAFLD, such that sole use of a simple OTC supplement is insufficient to overcome these liver deficiencies. With these results documenting that SAMe can have positive effects on basic measures of glucose homeostasis, future studies should pursue a more comprehensive evaluation of how SAMe interacts with multiple markers of Type 2 Diabetes. 

Microglia, as the immune cells of the brain, demonstrate profound dysfunction in chronic pathological conditions, such as obesity. In particular, hippocampal microglia are regionally distinct, such that they are more reactive, compared to cortical microglia, to immune-activating events [38]. Type 2 Diabetes increases hippocampal pro-inflammatory interferon-γ and Interleukin-1β levels along with activated microglia in Zucker fatty rats [39]. The decrease in hippocampal microglia density in DIO + SAMe mice was unexpected. Microglia respond to a variety of both pro- (interleukin-1β [IL-1β], tumor necrosis factor α [TNFα], etc.) and anti-inflammatory (interleukin-10 [IL-10]) cytokines. Obesity is associated with increased hippocampal levels of these pro-inflammatory factors, leading to activation and proliferation of hippocampal microglia [40]. Additionally, lipopolysaccharide (LPS) is an endotoxin that is increased by obesity (metabolic endotoxemia) [41] and may even drive diabetic phenotypes [42]. Twenty weeks of DIO increased the number of Iba1+ cells in the dentate gyrus, an effect driven by an increase in simple (i.e., fewer branched processes) microglia [43]. As people age, total CA1 microglia steadily increase, with microglial morphology increasingly resembling microglia in disease states such as Alzheimer’s, dementia, or encephalopathy [44]. In our results, all mice were older, while the number of microglia trended upward in DIO mice, but were amenable to a dramatic decrease in response to SAMe. Information on how SAMe affects factors related to microglial activity is limited, but macrophage studies provide some limited insight. SAMe reduces plasma TNFα levels in LPS-treated mice [45]. SAMe treatment to macrophages attenuates LPS-driven effects to increase NF-κB, IL-1β, IL-10, Nos2, and nitric oxide production [46]. SAMe also aids in the LPS-induced increase in IL-10 production in macrophage cell lines [47,48]. More established, is SAMe’s role as the primary methyl donor. Prolonged ingestion of SAMe increases brain SAMe levels [19] potentially affecting DNA methylation. Although increased methylation at promoters is known to decrease gene expression, methylation at other intragenic sites (introns, and exons) can enhance expression [49], as a result, SAMe can have both enhancing and diminishing effects on gene expression. Altered DNA methylation affecting expression of cytokines cannot be ruled out and may also be driving the effect of SAMe to increase TGFB1 transcripts in Chow mice. Previous studies have linked TGFβ1 to SAMe in cultured fibroblasts [50], lung macrophages [51], and hepatic stellate cells [52]. TGFβ1 is secreted by all cell types in the brain, although predominately by neurons [53]. It is necessary in mediating microglial quiescence, while suppression of TGFβ1 expression and signaling is a commonality across Alzheimer’s Disease (human and mouse models), amyotrophic lateral sclerosis, multiple sclerosis [10], and depression [54,55]. Restoration of TGFβ1-related signaling can attenuate amyloid beta, tumor necrosis factor alpha, and IL-6 levels in the hippocampus of APP/PS1 mice [56], and was found to be essential for (S)-ketamine’s and fluoxetine’s antidepressant effects in the hippocampus [54,55]. As such, therapeutics that increase TGFβ1 levels in the brain, and specifically the hippocampus, may be advantageous in limiting tissue damage (reviewed in [57]). The SAMe-induced increase in TGFβ1 corresponded to improved microglial morphology, albeit in the Chow mice only. This discrepancy suggests that TGFβ1 may control microglia through multiple pathways, some of which are more susceptible to impairment with obesity, and also warrants continued investigation into SAMe’s ability to modulate TGFβ1. Overall, these results support SAMe as a therapeutic for central inflammatory activity, its mechanisms of action however, remain poorly understood and warrant further investigation. 

Considerable work has shown that SAMe can improve cognitive outcomes; however, anxiety-like behaviors have been absent from the behavioral characterizations of its effects [22]. Obesity is associated with increased anxiety in clinical populations [58] and animal models [59]. Relative to untreated Chow mice, DIO mice demonstrated significantly decreased time spent in the center of the open field and longer latencies to enter the center. Although DIO mice were not as fast moving as Chow mice, and therefore covered less distance in the box, they were consistently mobile for the duration of the task, further validating the affective behaviors of DIO mice. SAMe did not affect behavior in Chow mice, presumably because they had no behavioral deficit related to anxiety. The increased anxiety-like behavior of DIO mice was modestly improved with SAMe with a significantly decreased latency to enter the center field in DIO mice. To our knowledge there is no known etiological difference between these open field measures, although both time in, and latency to enter, the center have been extensively used for decades [60]. Longer treatment duration or increased SAMe dose may produce a stronger anxiolytic effect, but the current dosing was not sufficient to ameliorate both measures of anxiety-like behavior in DIO mice; especially as some aspects of affective disorders have been linked to the dorsal hippocampus [61,62]. Chronic stress results in activation of microglia and recruitment of mononuclear cells, resulting in increased activated microglia in the dorsal hippocampus [8] and subsequent increased anxiety-like behavior in mice [63]. Furthermore, mice lacking hippocampal PHD and ring finger domaine 1 (PHRF1), which is essential for TGFβ1 signaling, displayed increased anxiety-like behavior [64]. SAMe’s effects to increase TGFβ1, improve branching and decrease the quantity of microglia in the dorsal hippocampus may have aided in reducing some anxiety-like behaviors in obese mice. Future work should also examine SAMe effects on ventral hippocampal microglia, as this area of the hippocampus also plays a prominent role in anxiety responses [62].

## 5. Conclusions

In summary, long-term supplementation with SAMe may provide some central and peripheral benefits. Although aged non-obese mice reaped superior benefits from SAMe, obese mice showed some improvements in hippocampal pathology and related anxiety behaviors. Currently, this study cannot determine whether the beneficial effects of SAMe were due to direct effects on the brain, or more indirectly by modulating peripheral sources of inflammation. Future studies should address this gap. With a longer treatment regimen, or as an adjuvant to exercise and diet changes, obese patients may benefit from taking this OTC supplement that carries no known side effects.

## Data Availability

The data presented in this study are available on request from the corresponding author.
